# Complete mitochondrial genome sequence of Asiatic lion (*Panthera leo persica*)

**DOI:** 10.1080/23802359.2016.1214541

**Published:** 2016-09-05

**Authors:** Wajeeda Tabasum, Sreenivas Ara, Niraj Rai, Kumarasamy Thangaraj, Ajay Gaur

**Affiliations:** a Conservation Genetics Lab, LaCONES, CSIR-Centre for Cellular and Molecular Biology, Hyderabad, India;; b Evolutionary Biology and Medical Genetics Lab, CSIR-Centre for Cellular and Molecular Biology, Hyderabad, India

**Keywords:** Asiatic lion, complete mitochondrial genome, ngs, conservation

## Abstract

The complete mitochondrial genome sequence 17,059 bp of Asiatic lion (*Panthera leo persica)* has been sequenced with the use of next generation sequencing technology using Ion Torrent ^PGM^ platform. The complete mitochondrial genome sequence of Asiatic lion consists of 13 protein-coding, 22 tRNA, and two rRNA genes, and 1 control region (CR). The mitochondrial genome is relatively similar to other felid mitochondrial genomes with respect to gene arrangement, composition, tRNA structures and skews of AT/GC bases to be typical of those reported for other mammals. The nucleotide composition of Asiatic lion mitogenome shows that there is more A-T% than G-C% on the positive strand as revealed by positive AT and CG skews. The overall base composition is 31.9% of A, 27.2% of C, 14.5% of G, and 26.2% of T. Most of the genes have ATA start codons, except ND1, COX2, ATP8, ATP6, ND4, and ND5 have ATG start codons.

The Asiatic lion (*Panthera leo persica*) is classified as a member of Felidae family consisting of two major sub families Pantherinae and Felinae. Pantherinae includes six big cats i.e. Tiger, Lion, Jaguar, Leopard, Snow leopard, and Clouded leopard (Johnson et al. 1996, 2006). Asiatic lions are the top carnivores and possess a prominent position atop the food chain in their only remaining natural habitat in Gir Forest of Gujarat in India, thus the reduction in their population size would lead to ecological imbalance (Donald & Kenneth 2010).

The blood sample used for DNA extraction and analysis was sampled from a male Asiatic lion named Siddharath with a studbook No. 370 from Nehru Zoological Park, Hyderabad, India (N: 17°21′04″, E: 78°26′59″).

In this study, the complete mitochondrial genome of Asiatic lion was sequenced and characterized. Two set of primers were designed based on highly conserved sequences of an alignment with full-length mitochondrial genomes from the available public database (unpublished) and used for PCR, with an average amplicon size of 8500 bp each with an overlap 614 bp were used for sequencing. The overall characteristics of mitogenome of Asiatic lion were identical to the typical vertebrate mitochondrial genome. The complete mitochondrial genome of Asiatic lion is 17,059 bp in length (GeneBank accession number KU234271), made up of 37 genes, which includes 13 protein-coding genes, 2 rRNA genes, and 22 tRNA genes, as well as a control region, is highly conserved among most vertebrates mitochondrial genome (Boore 1999).

Gene order and origin of reading frame of all protein-coding genes were identical to other members of Carnivora except for ND6 and nine tRNA genes. The total length of the 13 protein-coding genes was 11,364 bp, which corresponds to 66.62% of the mitochondrial genome sequence length. The longest gene was ND5 (1805 bp) and the shortest gene was ATP8 (195 bp). The start and stop codons appeared universal among species, except for ND2, COX1, COX3, ND3, ND4L, ND6, and CYTB (ATA start codon), the remaining protein-coding genes start with ATG. The length of the 22 tRNA genes ranges from 58 to 74 bp in Asiatic lion. In Asiatic lion, origin of L-strand replication (OL) was within a cluster of five tRNA genes i.e. tRNA^Trp^, tRNA^Ala^, tRNA^Asn^, tRNA^Cys^, and tRNA^Tyr^ and could also be folded into a stable stem-loop secondary structure. The noncoding region i.e. CR, was located between the tRNA-Pro and tRNA-Phe genes and is 1624 bps long.

The phylogenetic position of Asiatic lion was estimated using maximum-likelihood (ML) and the Bayesian inference (BI) (Figure 1) containing concatenated 13 protein-coding genes of 14 species derived from different families in suborder feliformia. The Bayesian analysis was performed using MRBAYES v 3.1.2 (Huelsenbeck et al. 2001) with four chains of 1.1 × 10^5^ generations and sampling the trees every 100 generations. ML tree was similar to the BI tree. The monophyly of genus *panthera* was clearly depicted and were statistically supported by high bootstrap values.

**Figure 1.**
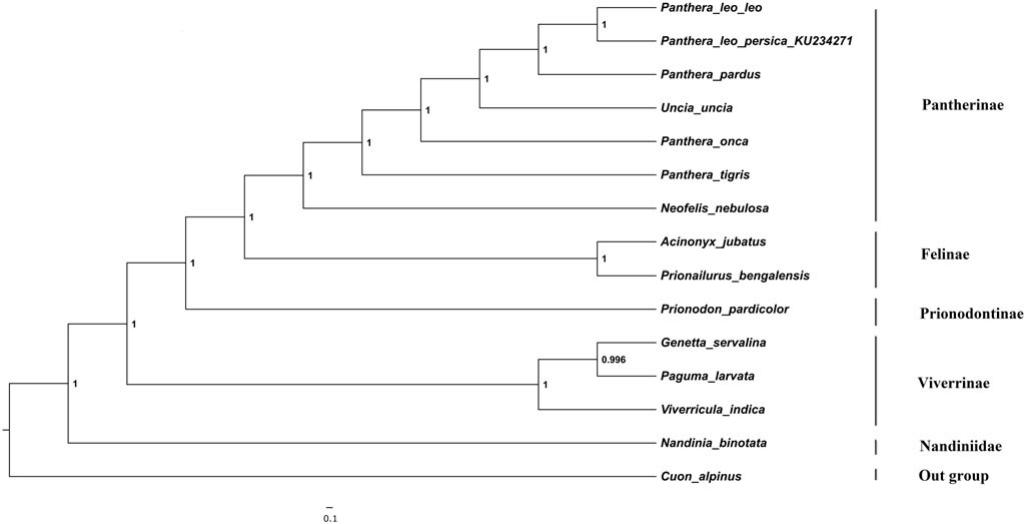
The Bayesian majority-rule consensus tree is inferred from the combined data set of 13 mitochondrial protein-coding genes. The node value represents the Bayesian posterior probabilities. The phylogenetic tree was rooted using *Cuon alpines* (NC_013445). Our sample sequence was *Panthera leo persica* (KU_234271). The analysed species and the corresponding NCBI accession numbers are as follows: *Panthera leo leo* (KF776494), *Panthera pardus* (EF_551002), *Uncia uncia* (NC_010638), *Panthera onca* (KM_236783), *Panthera tigris* (EF_551003), *Neofelis nebulosa* (NC_008450), *Acinonyx jubatus* (NC_005212), *Prionailurus bengalensis* (KP_246843), *Nandinia binotata* (NC_024567), *Viverricula indica* (NC_025296), *Genetta servalina* (NC_024568), *Paguma larvata* (NC_029403) and *Prionodon pardicolor* (NC_024569).

The characterization of Asiatic lion mitogenome will contribute to more refined phylogeny of big cats. Our study will also facilitate concentrated efforts towards the conservation and proper management of the only remaining population of Asiatic lions in India.

## Nucleotide sequence accession number

The complete genome sequence of Asiatic lion (*Panthera leo persica)* has been assigned and Genbank accession number KU234271.
